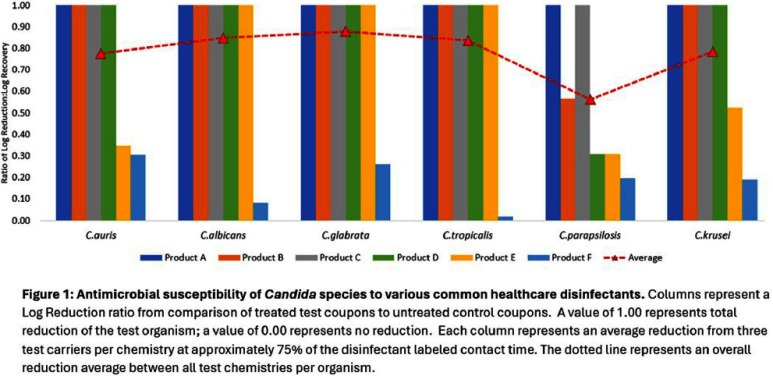# Antimicrobial Susceptibility Variation Among Emerging WHO Listed Candida Pathogens

**DOI:** 10.1017/ash.2025.331

**Published:** 2025-09-24

**Authors:** Joshua Luedtke, Teresa Podtburg, Lisa Hellickson, Kris Owens, Karoline Sperling, Joseph Wegner

**Affiliations:** 1 Ecolab

## Abstract

**Background:** Invasive fungal diseases (IFDs) are severe infections caused by fungi that can spread throughout the body, particularly in individuals with weakened immune systems. These infections are increasingly challenging, especially those that are resistant to antifungal treatments. IFDs represent an emerging global threat, highlighting the urgent need for increased attention and research. In 2022, the World Health Organization (WHO) released the WHO Fungal Priority Pathogen List (WHO-FPPL), ranking 19 fungal pathogens as critical, high, or medium based on criteria such as incidence, treatment options, and mortality rates. While Candida albicans and Candida auris are well-known and included on the list, it also features four other species of concern (C. glabrata, C. tropicalis, C. parapsilosis, C. krusei) that are not typically listed on disinfectant master labels. This study aims to evaluate the antimicrobial susceptibility differences between Candida species from commonly used healthcare disinfectant products. **Method:** Antimicrobial efficacy testing was conducted using common healthcare disinfectants against the WHO-FPPL listed Candida species, following standard operating procedures typically required for disinfectant product registration by the U.S. EPA. Each disinfectant formulation represented a common active ingredient, or active ingredient blend, used in healthcare settings for surface disinfection, ranging from ready-to-use sprays to wipes. Products were tested at approximately 75% of the manufacturer-defined contact time listed on the EPA master label to stress the chemistry and elucidate antimicrobial susceptibility differences between Candida species. **Results:** Antimicrobial efficacy varied across Candida species for the tested chemistries. Of the six Candida species tested, C. parapsilosis was the most difficult to eradicate. The remaining Candida species exhibited less variability with C.auris and C. krusei demonstrating slightly lower susceptibility across all of the disinfectant types than C.albicans, C. glabrata, and C. tropicalis. **Conclusion:** This study underscores the variability in efficacy between these emerging fungal pathogens and common healthcare disinfectants. While C. auris remains a primary concern in healthcare settings, these findings highlight the continued need for ongoing fungal surveillance, research of emerging fungi, and the potential impact on environmental hygiene practices.

**Figure:**